# Pre-diabetes, Diabetes Mellitus and Related Cardio-metabolic Risk Factors in the Southern Coastal Region of Iran Middle-aged and Elderly Population; Bandare-Kong Cohort Study

**DOI:** 10.34172/aim.2022.68

**Published:** 2022-07-01

**Authors:** Zahra Ghaemmaghami, Ebrahim Eftekhar, Roghayeh Shahbazi, Azim Nejatizadeh, Mehdi Shahmoradi, Masoumeh Kheirandish

**Affiliations:** ^1^Rajaie Cardiovascular Medical and Research Center, Iran University of Medical Sciences, Tehran, Iran; ^2^Endocrinology and Metabolism Research Center, Hormozgan University of Medical Sciences, Bandar Abbas, Iran; ^3^Cardiovascular Research Center, Hormozgan University of Medical Sciences, Bandar Abbas, Iran; ^4^Cellular and Molecular Medicine Department, Faculty of Medicine, University of Ottawa, Ottawa, Canada; ^5^Molecular Medicine Research Center, Hormozgan Health Institute, Hormozgan University of Medical Sciences, Bandar Abbas, Iran

**Keywords:** Diabetes mellitus, Impaired fasting glucose, PERSIAN Cohort Study, Prevalence

## Abstract

**Background:**

To evaluate the prevalence of type 2 diabetes mellitus (T2DM), impaired fasting glucose (IFG), and its cardio-metabolic risk factors in the southern Iranian adult population.

**Methods:**

This is a population-based cross-sectional survey on 3944 middle-aged and elderly adults (35–70 years) from Bandare-Kong. The participants were recruited from 2016 to 2018 and the first phase data of the Bandare-Kong Cohort as a part of the PERSIAN Cohort were used for analysis.

**Results:**

Among the 3944 included adults, the age-adjusted prevalence of T2DM and IFG was 17.40% and 20.61%, respectively. Mean FPG was higher among those older than 55 years, females, rural residents, current cigarette smokers, hypertriglyceridemia, hypercholesterolemia, unemployed and low educational level in subjects with diabetes and pre-diabetes. T2DM and IFG were more prevalent in women and men, respectively. Also, those with higher waist circumference (WC), higher body mass index (BMI), lower educational levels, rural residents, former cigarette smokers, hypertension (HTN), hypercholesterolemia, hypertriglyceridemia and age older 45 years, had a higher T2DM and IFG prevalence. Multivariable regression analysis showed that older age, higher WC, HTN and hypertriglyceridemia and living in rural regions were statistically significant predictors of T2DM and pre-diabetes while BMI≥25 kg/m^2^ was the only significant risk factor for IFG.

**Conclusion:**

The current study illustrated that T2DM and IFG have a high prevalence among the middle-aged and elderly adult Iranian population, particularly in rural dwellers. Hence, prevention strategies should be implemented to reduce diabetes and pre-diabetes, especially in rural areas.

## Introduction

 Type 2 diabetes mellitus (T2DM) is a chronic and complicated illness that has a major impact on personal wellbeing and the health care system economy. Different factors such as population growth aging, sedentary lifestyle, increasing prevalence of obesity, high-calorie dietary intake, and other unknown factors have increased the prevalence of T2DM and pre-diabetes. Hence, earlier diagnosis of T2DM and pre-diabetes improves life expectancy and quality of life.^1^ Based on the estimation of the World Health Organization (WHO), T2DM will become the seventh leading cause of death by 2030.^2^

 The prevalence of diabetes in adults aged 20–79 years was estimated at 8.8% in 2015 and is projected to reach 10.4% in 2040 worldwide. The regions that are projected to experience the highest growth rates regarding the number of people with diabetes are Africa (140.7% increase by 2040), the Middle East, and North Africa (103.8% increase by 2040).^3^ The prevalence of T2DM has a geographical and ethnic-cultural diversity.^4^ A high prevalence of undiagnosed T2DM has been reported in a recent meta-analysis in Iran.^5^ In addition, a T2DM prevalence rate of 12.3% has previously been reported among middle-aged Iranian adults.^6^

 There are limited data regarding diabetes epidemiology in the southern coastal area of Iran; therefore, we aimed to evaluate the prevalence of T2DM and pre-diabetes as well as investigate their associated cardio-metabolic risk factors in Bandare-Kong city in southern Iran.

## Materials and Methods

 The current study is the first phase of Bandare-Kong Non-Communicable Disease (BKNCD) Cohort Study in a coastal area in the Hormozgan province, southern Iran, which is part of the PERSIAN (Prospective Epidemiological Research Studies in IrAN) cohort study. The study methods have been described previously.^7^ From November 17, 2016 to November 22, 2018, a total of 4063 individuals aged 35-70 years were recruited. Incomplete records and pregnant women were excluded, leaving 3944 individuals for the final analysis.

 Sociodemographic factors including age, gender, marital status, education level, employment status, place of residence, cigarette smoking, wealth score index (WSI), and physical activity were recorded by trained staff through a face-to-face interview.

 Weight was measured with minimum clothing and without shoes by a mechanical scale (measurement accuracy of 0.5 kg). Height was measured with the subjects standing shoeless and their shoulders placed normally. The circumference at the mid-point of iliac crest and the last palpable rib was taken as waist circumference (WC). WC was measured twice for each subject and the average of two measurements was recorded. A stretch-resistant measuring tape was used for all measurements. The accuracy of the recorded values was to the nearest 0.5 cm. By dividing WC to hip circumference (HC), the waist-to-hip ratio (WHR) was estimated to the nearest 0.01.

 To calculate body mass index (BMI), weight (kg) was divided by the square of height (m). Based on WHO guidelines, underweight was defined as BMI < 18.5 kg/m^2^, normal weight as 18.5 ≤ BMI < 25, overweight as 25 ≤ BMI < 30, and obesity as BMI ≥ 30.^8^

 A standard mercury sphygmomanometer with individualized cuff size was used to measure blood pressure (BP). All BP measurements were done after 5 minutes of rest while the subjects were seated, their arms were at heart level, and their feet were on the floor.^9^ BP was measured twice at least 5 min apart and the average was recorded. All BP measurements were made on the right arm by a trained nurse. BP was measured a third time if there was a greater than 10-mmHg difference between the two systolic and/or diastolic readings. In this case, the two readings with the least difference were recorded. Subjects were considered hypertensive in case of self-reported hypertension (HTN), taking anti-hypertensive drugs, or newly diagnosed HTN. New cases of HTN were diagnosed when systolic blood pressure (*SBP*) ≥ 140 mm Hg and/or diastolic blood pressure (DBP) ≥ 90 mm Hg were present.

 Fasting plasma glucose (FPG) and the lipid components, including triglyceride (TG), total cholesterol (TC), high-density lipoprotein cholesterol (HDL-C) and low-density lipoprotein cholesterol (LDL-C) were measured in venous blood samples following overnight 8-hour and 12-hour fasting on a separate day, respectively using the enzymatic methods. The following criteria have been proposed by the American Diabetes Association (ADA) for T2DM: FPG ≥ 126 mg/dL or using anti-hyperglycemic agents and impaired fasting glucose (IFG) as 100 ≤ FPG < 126 mg/dL.^10^

 Hypercholesterolemia, by definition, is serum TC > 200 mg/mL or anti-lipid drug consumption. Hypertriglyceridemia is considered if TG > 150 mg/dL or taking anti-lipid drugs.^11^

 According to the Iranian obesity association guideline, the cut-off value of high WC is WC ≥ 95 cm for both men and women.^12^

 Cigarette smoking status was based on self-reported data. Current cigarette smoking was attributed to individuals who were current smokers or who had smoked at least 100 cigarettes in their lifetime. Ex-cigarette smokers were those who had smoked at least 100 cigarettes in their lifetime but with no history of smoking since six months ago.^13^

 Physical activity score was recorded as 24-hours activities including work, exercise, and leisure time activities calculated on a weekly basis. It was categorized as low-, moderate-, and vigorous-intensity.^14^

 The WSI was separately estimated by multiple correspondence analysis of the variables. The analysis was performed for the following variables: access to a freezer, a washing machine, a dishwasher, a computer, internet, a motorcycle, a vacuum cleaner, color TV, owning a cell-phone, a personal computer or a laptop, and international trips in a lifetime. According to their total asset score, subjects were ranked and then categorized into five quintiles, including very rich, rich, average, poor, and very poor.

 Quantitative and qualitative categorized data were shown as means and standard deviations, proportions, and frequencies, respectively. Since the BKNCD is a study of large data, an assumption of normally distributed variables was made. The estimated prevalence of diabetes and pre-diabetes with 95% confidence intervals (CIs) was calculated. In addition, the prevalence was standardized by the direct standardization method to overcome the confounding effects of age and sex. The qualitative variables, quantitative variables association analysis between two and more independent categories were analyzed by chi-square test, independent *t *test, and analysis of variance (ANOVA), respectively. Finally, binary logistic regressions were performed to evaluate the effect of multiple variables on T2DM and pre-diabetes prevalence. All the potential factors with *P* values ≤ 0.2 in univariable correlations, were simultaneously included in the multivariable logistic regression model using the “Wald” method. For instance, diabetes and pre-diabetes groups were coded 1, and the healthy state was coded 0. Therefore, the odds of each diabetes and pre-diabetes state were reported compared to the healthy state. A cutoff point of *P* value less than 0.05 was considered as the significance level. The analyses were carried out using the SPSS software version 22.0.

## Results

 Among the 3944 study participants, approximately 40% of the population had abnormal blood glucose levels. Men comprised 1701 (43.1 %) of the total population (Table 1). Table 2 provides information about the age--standardized prevalence (ASP) of T2DM and IFG. According to Table 2, the ASP of T2DM and IFG were 17.4% and 20.6%, respectively. T2DM was more prevalent in women than men (21.7% versus 15.9%). Contrary to T2DM, pre-diabetes was more common in males, (23.5% versus 19.2%).

**Table 1:** Prevalence of IFG and T2DM, Bandare-Kong Cohort Study (n = 3944)

**Glycemic Status**	**Men, No. (%)**	**Women, No. (%)**	**Total, No. (%)**
Normal	1030 (60.6)	1325 (59.1)	2355 (59.7)
IFG	400 (23.5)	431 (19.2)	831 (21.1)
T2DM	271 (15.9)	487 (21.7)	758 (19.2)
Total	1701 (43.1)	2243 (56.9)	3944 (100.0)

IFG, impaired fasting glucose; T2DM, type 2 diabetes mellitus.

**Table 2:** Crude and ASP of T2DM and IFG based on Sex: Bandare-Kong Cohort Study (*n* = 3944)

**Glycemic status**	**Total population**		**Men**		**Women**	
	**Crude (95% CI)**	**ASP (95% CI)**	**Crude (95% CI)**	**ASP (95% CI)**	**Crude (95% CI)**	**ASP (95% CI)**
IFG	21.07 (19.81–22.38)	20.61 (19.35–21.88)	23.52 (21.52–25.60)	23.32 (21.29–25.35)	19.22 (17.60–20.90)	18.57 (16.96–20.17)
T2DM	19.21 (18.00–20.48)	17.40 (16.30–18.50)	15.93 (14.22–17.76)	14.54 (12.95–16.13)	21.71 (20.02–23.48)	19.57 (18.06–21.08)

ASP, Age-standardized prevalence by census data; CI, confidence interval; IFG, impaired fasting glucose; T2DM, type 2 diabetes mellitus.

Table 3 shows the mean FPG level and the prevalence of T2DM and pre-diabetes by different cardio-metabolic and socio-economic risk factors. As shown in Table 3, the mean FPG levels were significantly higher in pre-diabetic and diabetic subjects, those older than 55 years, women, participants with hypercholesterolemia and hypertriglyceridemia, rural residents, the unemployed, participants who were single, widowed, and divorced, current cigarette smokers, and individuals with lower educational level. The IFG participants with high WC, higher BMI, and hypertensive people had higher mean FPG compared to their counterparts. On the other hand, among T2DM, greater mean FPG levels pertained to single marital status, normal WC, normal BMI, and normotensive groups. Notwithstanding, pre-diabetic subjects, who had BMI < 25 kg/m^2^, WC < 95 cm and normotensive also had a higher mean of FPG.

**Table 3:** Mean Fasting Plasma Glucose Level and Prevalence of Diabetes and Pre-diabetes Based on Different Independent Variables, Bandare-Kong Cohort Study, Iran (n = 3944)

**Variables**	**FPG (mg/d**L**) Mean (SD)**			***P*** **Value**	**Prevalence**		***P*** **Value**
	**Normal **	**IFG **	**T2DM**		**IFG **	**T2DM **	
Age categories years %^*^					CI (95%)	CI (95%)	< 0.001
35–44	88.07 (87.67–88.47)	106.21 (105.53–106.90)	170.52 (157.14–183.89)	< 0.001	18.6 (16.7–20.5)	8.1 (6.8–9.5)	
45–54	89.14 (88.65–89.64)	107.60 (106.86–108.34)	166.78 (158.54–175.01)	< 0.001	23.0 (20.6–25.4)	20.4 (18.1–22.7)	
55–70	89.26 (88.65–89.88)	108.44 (107.64–109.23)	175.08 (168.49–181.67)	< 0.001	22.6 (20.2–25.2)	34.3 (31.5–37.1)	
Gender %							< 0.001
Male	88.87 (88.44–89.29)	107.10 (106.48–107.71)	168.52 (161.00–107.47)	< 0.001	23.5 (21.5–25.6)	15.9 (14.2–17.7)	
Female	88.43 (88.06–88.80)	107.58 (106.99–108.18)	173.34 (167.08–179.59)	< 0.001	19.2 (17.6–20.9)	21.7 (20.0–23.4)	
BMI (kg/m^2^)%							< 0.001
< 25	87.81 (87.37–88.26)	106.75 (106.00–107.50)	186.79 (177.03–196.55)	< 0.001	17.5 (15.5–19.5)	14.1 (12.3–15.9)	
≥ 25	89.20 (88.85–89.56)	107.61 (107.09–108.13)	165.86 (16.39–171.33)	< 0.001	23.3 (21.6–25.0)	21.5 (19.9–23.1)	
WC (cm) %							< 0.001
< 95	88.08 (87.72–88.45)	106.77 (106.20–107.35)	177.17 (169.52–184.83)	< 0.001	18.8 (17.1–20.5)	14.2 (12.7–15.7)	
≥ 95	88.48 (89.05–89.90)	107.91 (107.28–108.54)	167.40 (161.16–173.63)	< 0.001	24.0 (22.0–26.1)	24.3 (22.3–26.3)	
Place of residence %							< 0.001
Urban	88.41 (88.12–88.71)	107.21 (106.73–107.70)	168.94 (168.66–174.21)	< 0.001	19.4 (18.0–20.7)	18.3 (16.9–19.6)	
Rural	90.24 (89.41–91.06)	107.83 (106.92–108.74)	184.38 (172.57–196.18)	< 0.001	31.6 (27.8–35.5)	21.8 (18.5–25.4)	
Marital status %							0.024
Single	86.64 (84.90–88.37)	107.64 (104.36–110.92)	196.80 (136.98–256.62)	< 0.001	23.7 (15.4–33.6)	10.8 (5.2–18.8)	
Married	88.68 (88.38–88.97)	107.24 (106.80–107.69)	170.50 (165.41–175.60)	< 0.001	21.0 (19.6–22.4)	18.5 (17.2–19.8)	
Widowed + Divorced	88.66 (87.69–89.62)	108.42 (106.75–110.10)	176.18 (160.11–192.26)	< 0.001	22.2 (17.7–27.1)	24.1 (19.4–29.1)	
Occupation %							< 0.001
Employed	88.74 (88.34–89.15)	106.94 (106.33–107.55)	168.08 (160.44–175.72)	< 0.001	22.3 (20.3–24.3)	14.0 (12.4–15.7)	
Unemployed	88.51 (88.13–88.90)	107.71 (107.12–108.31)	173.36 (167.20–179.52)	< 0.001	20.3 (18.6–22.0)	22.7 (20.9–24.5)	
Smoking status %							0.006
Former	89.11 (87.95–90.26)	105.54 (104.25–106.83)	164.25 (147.21–181.29)	< 0.001	21.5 (16.3–27.5)	23.8 18.3–29.9)	
Current	87.74 (86.83–88.64)	107.64 (105.88–109.40)	173.20 (154.29–192.10)	< 0.001	19.0 (15.0–23.4)	13.3 (9.9–17.3)	
Never	88.74 (88.44–89.04)	107.44 (106.98–107.91)	171.27 (166.04–176.51)	< 0.001	21.4 (19.9–22.8)	19.2 (17.8–20.6)	
Hypertension %							< 0.001
Yes	89.04 (88.48–89.60)	108.55 (107.82–109.28)	169.39 (163.31–175.46)	< 0.001	23.6 (21.2–26.0)	33.7 (31.1–36.3)	
No	88.50 (88.18–88.82)	106.67 (106.15–107.19)	174.68 (166.79–182.56)	< 0.001	19.9 (18.4–21.4)	12.3 (11.1–13.6)	
Hypercholesterolemia %							< 0.001
Yes	88.49 (89.13–89.85)	107.64 (107.09–108.20)	172.92 (167.40–178.45)	< 0.001	22.4 (20.7–24.2)	23.9 (22.1–25.7)	
No	87.71 (87.29–88.13)	106.90 (106.23–107.57)	168.02 (158.15–177.88)	< 0.001	19.1 (17.4–21.2)	13.0 11.4–14.6)	
Hypertriglyceridemia %							< 0.001
Yes	89.65 (89.14–90.16)	107.43 (106.77–108.09)	172.25 (166.53–177.97)	< 0.001	24.4 (22.2–26.6)	31.7 (29.3–34.1)	
No	88.24 (87.91–88.57)	107.29 (106.73–107.85)	170.48 (161.74–179.21)	< 0.001	19.1 (17.5–20.7)	11.8 (10.5–13.1)	
Education (years) %							< 0.001
< 6	88.70 (88.32–89.09)	107.85 (107.30–108.39)	175.54 (169.74–181.34)	< 0.001	22.2 (20.5–23.9)	22.9 (21.2–24.6)	
6–12	88.55 (88.10–89.01)	106.51 (105.72–107.29)	164.32 (154.83–173.81)	< 0.001	19.3 (17.1–21.6)	13.7 (11.8–15.7)	
≥ 12	88.43 (87.57–89.29)	106.59 (105.22–107.96)	135.15 (119.78–150.52)	< 0.001	21.0 (18.6–25.8)	8.3 (5.5–11.9)	

CI, confidence interval; IFG, impaired fasting glucose; T2DM, type 2 diabetes mellitus; BMI, body mass index; WC, waist circumference; FPG, fasting plasma glucose. *% indicates the prevalence of diabetes and prediabetes in different sub-categories of independent variables.

 T2DM and IFG were more prevalent among higher WC, higher BMI, lower educational levels, rural residents, former cigarette smokers, HTN, hypercholesterolemia and those with hypertriglyceridemia. Whereas T2DM was more prevalent in women, widowed and divorced, unemployed people, and older adults ( ≥ 45 years), IFG was more common among men, single, and middle-aged 45-54 year-old adults.

 The crude and adjusted odds ratios of different related risk factors by multivariable logistic regression have been presented in Tables 4 and 5. The results illustrated that women are nearly 50% more at risk for T2DM than men (odds ratio [OR] = 1.48, 95% CI: 1.22–1.78); however, the risk of pre-diabetes in women is about 20% lower than men (OR = 0.8, 95% CI: 0.68–0.96). According to the results, subjects older than 55 years had OR = 3.87, 95% CI: 3.04–4.94, and OR = 1.81, 95% CI: 1.45–2.25 for T2DM and pre-diabetes, respectively. Central obesity (T2DM: OR = 1.37, 95% CI: 1.14–1.65; IFG: OR = 1.24, 95% CI:1.78–2.74), HTN (T2DM: OR = 2.18, 95% CI: 1.81–2.62, IFG: OR = 1.31, 95% CI: 1.09–1.59), rural area residents (T2DM: OR = 1.47, 95% CI: 1.13–1.92; IFG: OR = 2.21, 95% CI: 1.78–2.74) and hypertriglyceridemia (T2DM: OR = 2.91, 95% CI: 2.43–3.49; IFG: OR = 1.65, 95% CI: 1.39–1.97) were other predictors. While BMI as a risk factor had no significant association with T2DM after multivariable regression analysis, the pre-diabetes condition was more prevalent in subjects with BMI ≥ 25 kg/m^2^ (OR = 1.42, 95% CI: 1.13–1.77). Furthermore, intense physical activity reduced T2DM odds by 50%.

**Table 4:** Univariate Logistic Regression Analysis of Diabetes and Pre-diabetes by Independent Variables, Bandare-Kong Cohort Study, Iran (n = 3944)

**Variables**	**Diabetes (n=758)**		**Pre– diabetes (n=831)**	
	**Crude OR**	***P*** ** Value**	**Crude OR**	***P*** ** Value**
Age categories (y)				
35–44	Ref		Ref	
45–54	3.29 (2.61–4.15)	< 0.001	1.60 (1.33–1.93)	< 0.001
55–70	7.26 (5.80–9.11)	< 0.001	2.07 (1.70–2.53)	< 0.001
Gender				
Male	Ref		Ref	
Female	1.39 (1.18–1.65)	< 0.001	0.83 (0.71–0.98)	0.029
BMI (kg/m^2)^				
< 25	Ref		Ref	
≥ 25	1.89 (1.58–2.27)	< 0.001	1.65 (1.39–1.96)	< 0.001
WC (cm)				
< 95	Ref		Ref	
≥ 95	2.21 (1.87–2.62)	< 0.001	1.65 (1.41–1.94)	< 0.001
Place of residence				
Urban	Ref		Ref	
Rural	1.24 (1.01–1.54)	0.044	2.18 (1.77–2.69)	< 0.001
Marital status				
Single	Ref		Ref	
Married	1.88 (0.97–3.65)	0.060	0.96 (0.58–1.57)	0.881
Widowed + Divorced	2.73 (1.32–5.61)	0.006	1.14 (0.65–2.00)	0.637
Occupation				
Employed	Ref		Ref	
Unemployed	1.81 (1.52–2.12)	< 0.001	0.98 (0.83–1.15)	0.831
Smoking status				
Never	Ref		Ref	
Current	0.64 (0.46–0.88)	0.007	0.78 (0.58–1.03)	0.086
Former	1.31 (0.95–1.80)	0.098	1.09 (0.77–1.54)	0.608
Hypertension				
No	Ref		Ref	
Yes	4 (3.67–5.19)	< 0.001	1.88 (1.58–2.23)	< 0.001
Hypercholesterolemia				
No	Ref		Ref	
Yes	2.34 (1.96–2.80)	< 0.001	1.47 (1.25–1.73)	< 0.001
Hypertriglyceridemia				
No	Ref		Ref	
Yes	4.25 (3.57–5.05)	< 0.001	2.01 (1.70–2.37)	< 0.001
Education (y)				
< 6	Ref		Ref	
6–12	0.49 (0.40–0.59)	< 0.001	0.71 (0.59–.85)	< 0.001
≥ 12	0.28 (0.18–0.42)	< 0.001	0.73(0.55–0.98)	0.037
WSI				
Very poor	Ref		Ref	
Poor	1.00 (0.78–1.27)	0.974	0.83(0.65–1.07)	0.154
Average	0.91 (0.71–1.17)	0.474	0.93(0.72–1.19)	0.578
Rich	0.91 (0.71–1.17)	0.492	0.87(0.68–1.11)	0.273
Very rich	0.70 (0.54–0.91)	0.009	0.88(0.69–1.13)	0.340
Physical activity score (METs)				
Low (24–36.5)	Ref		Ref	
Moderate (36.6–44.9)	0.58 (0.486–0.69)	< 0.001	0.96(0.79–1.16)	0.705
Vigorous ( ≥ 45)	0.40 (0.30–0.53)	< 0.001	0.86(0.66–1.11)	0.263

BMI, body mass index; WC, waist circumference; WSI, wealth score index; OR, odds ratio; Reference is normoglycemic participants.

**Table 5:** Multivariable Logistic Regression Analysis of Diabetes and Pre-diabetes by Independent Variables, Bandare-Kong Cohort Study, Iran (n = 3944)

**Variables**	**Diabetes**		**Pre–diabetes**	
	**Adjusted OR**	***P*** ** Value**	**Adjusted OR**	***P*** ** Value**
Age categories (y)				
35–44	Ref		Ref	
45–54	2.29 (1.79–2.92)	< 0.001	1.49 (1.22–1.81)	< 0.001
55–70	3.87 (3.04–4.94)	< 0.001	1.81 (1.45–2.25)	< 0.001
Gender				
Male	Ref		Ref	
Female	1.48 (1.22–1.78)	< 0.001	0.81 (0.68–0.96)	0.016
BMI (kg/m^2^)				
< 25	—		Ref	
≥ 25	—		1.42 (1.13–1.77)	0.002
WC (cm)				
< 95	Ref		Ref	
≥ 95	1.37 (1.14–1.65)	0.001	1.24 (1.01–1.53)	0.041
Place of residence				
Urban	Ref		Ref	
Rural	1.47(1.13–1.92)	0.004	2.21 (1.78–2.74)	< 0.001
Hypertension				
No	Ref		Ref	
Yes	2.18 (1.81–2.62)	< 0.001	1.31 (1.09–1.59)	0.004
Hypertriglyceridemia				
No	Ref		Ref	
Yes	2.91 (2.43–3.49)	< 0.001	1.65 (1.39–1.97)	< 0.001
Physical activity score (METs)				
Low (24–36.5)	Ref			
Moderate (36.6–44.9)	0.69(0.57–0.84)	< 0.001	––––	––––
Vigorous ( ≥ 45)	0.59(0.43–0.84)	0.001	––––	––––

BMI, body mass index; WC, waist circumference; OR, odds ratio; Reference is normoglycemic participants.

 The non-smokers and ex-smoker had higher mean FPG levels and a higher prevalence of T2DM and IFG than the current smokers, and the T2DM risk was significantly reduced in participants who had WSI in the very rich group (OR: 0.70 (0.54–0.91), but the association was not significant after multivariable regression analysis.

## Discussion

 We found a relatively high prevalence of diabetes and pre-diabetes in Bandare-Kong residents; approximately half of our population were suffering from T2DM and IFG. Age > 55 years, female gender, WC ≥ 95 cm, rural residence, HTN, and hypertriglyceridemia were associated with a higher prevalence of IFG and diabetes. BMI ≥ 25 kg/m^2^ was an independent predictor of the higher prevalence of IFG, while moderate and vigorous physical activity reduced progression to T2DM. Although the risk of diabetes decreased in current cigarette smokers compared to non-smokers and ex-smokers in the initial analysis, the difference did not remain significant after multivariable adjustment analysis.

 In our population, the prevalence of T2DM was approximately 1.5-folds higher compared to similar studies in different parts of Iran.^6,15^ For instance, the current study revealed higher prevalence rates of T2DM compared to the study by Ebrahimi et al The prevalence of T2DM in men, women, and the total population of their study was 10.4%, 13.6%, and 12.3%, respectively.^6^ Disparate T2DM definition criteria could explain the difference between studies. They regarded individuals as having T2DM if the random blood glucose levels were greater than 200 mg/dL and/or if they were using blood glucose-lowering drugs, while in the current study, those who had abnormal FPG and/or self-reported T2DM and/or were receiving treatment were diagnosed as T2DM. Another study in southern Iran showed a 9.9% prevalence for T2DM with higher prevalence in women compared to men (11.9% vs. 7.6%).^16^

 The prevalence rates of diabetes and pre-diabetes in our study were also higher than the rates reported by two large population-based surveys in Iran. The prevalence of T2DM and IFG was reported as 7.7% and 16.8%, respectively in the study by Esteghamati et al on 70,981 Iranians aged 25-64 years.^17^ Hadaegh et al evaluated 9,489 participants in the Tehran Lipid and Glucose Study and reported an age-standardized prevalence of 7.3%, 6.7%, 4.2%, 4.9%, and 9.1% for isolated IFG, isolated impaired glucose tolerance (IGT), IFG/IGT, undiagnosed diabetes, and known diabetes, respectively.^18^ The most probable explanation for higher figures in our study compared to these two studies can be different geographical areas and lifestyles; we included people who live in a coastal city in the south. On the other hand, the worldwide increasing trend of T2DM and IFG, together with the time gap between these two large studies and ours as well as lifestyle change in Iranian people could be other reasons for these discrepancies.

 By comparing our results to two nearby Middle Eastern countries, we found that again, the prevalence of diabetes and pre-diabetes was higher in our study. T2DM prevalence was 11.0% in Turkey,^19^ and 4.5% in Saudi Arabia.^20^ This difference between the Turkish study and ours can be due to a nearly 20-year interval between the studies; however, the study in Saudi Arabia has been conducted more recently. Here, the lower rate can be justified by only including individuals with > 200 mg/dL postprandial glucose levels and missing those taking drugs or individuals having high FPG.^20^

 With regard to age, our results in people over 55 years of age were similar to the study of Tanjani et al,^21^ which shows the importance of attention to T2DM control in this age group. Other studies have also demonstrated increased prevalence of T2DM with advancing age.^17,18,20^

 In the current study, the prevalence of T2DM was 15.9% in men and 21.7% in women. Moreover, diabetes mellitus was more prevalent among women in all age categories, while IFG was more prevalent in men. In multivariable regression analysis, female gender increased the risk of diabetes by nearly 50% which is in agreement with previous Iranian studies^16,17,22^ and the National Health and Nutrition Examination Survey.^23^ The Yazd health study also reported a higher diabetes prevalence among women, although women comprised a more significant proportion of pre-diabetes in their study.^22^ This might be explained by variations in the demographic features of study populations. In general, a significant sex difference has been observed with respect to fasting glucose levels and response to an oral glucose tolerance test. IFG has been shown to be more prevalent in men and IGT in women. The reasons for this include lower skeletal muscle mass and higher adipose tissue mass in women, as well as the effects of gonadal hormones, postmenopausal hormone therapy with estrogens that could promote insulin resistance and IGT in women compared to men of the same age. On the other hand, the prevalence of T2DM is also affected by gender. It depends on the reproductive life stage; diabetes is more common in men after puberty while it is more frequent in women at older age and in the post-menopausal stage.^24^ Of note, nearly 60% of women older than 45 years in our study were in the menopausal stage (data not shown). Furthermore, the older age and the higher BMI and WC in the women of our study compared to men can be responsible for the higher prevalence of diabetes and pre-diabetes in females. Another explanation can be lower physical activity and educational level in women as we found a higher prevalence of DM in people with lower educational levels.

 The most concerning result of our study was the high prevalence of diabetes and IFG among rural residents. We found that the prevalence of DM and IFG was 21.8% and 31.6% in rural residents, respectively. This is in line with previous studies.^25-27^ Also, the rural dwellers in our study were older, had poor education, were mostly unemployed, had a higher frequency of HTN, and had a poor wealth index compared to urban residents (data not shown). The factors mentioned above are important risk factors for diabetes and pre-diabetes and can explain the higher prevalence of DM and IFG in rural residents in our study. In addition, the rural community may face challenges in terms of healthy food consumption, fewer health care facilities, and transportation that can contribute to their poor health condition. Nevertheless, based on the other nationwide studies in Iran, DM has been reported to be more prevalent in urban areas.^28^

 The relationship between educational status and diabetes prevalence in our study is similar to other studies in Iran and other countries which have demonstrated that people with a substandard education have a 28% higher chance of developing diabetes.^6,29,30^ This relationship may be due to greater attention to the healthy lifestyle in individuals with higher education which is an important determinant of public health and prevalence of T2DM.

 Our multivariable analysis also showed that higher WC increased the risk of diabetes and pre-diabetes. Similarly, other studies in Iran and other countries have found similar results,^6,31^ showing a strong justification for weight loss recommendations and that the risk of diabetes can be lowered with decreased central fat. We also found that HTN and hypertriglyceridemia increase the risk of T2DM and pre-diabetes. This can be expected regarding the role of these variables in increasing insulin resistance.^32^

 Some of the current study’s limitations are as follows: First of all, its cross-sectional design made it impossible to determine an actual cause and effect association because the exposure and outcome are evaluated concurrently. Secondly, it is a population-based study conducted in the middle-aged and elderly in southern Iran; therefore, the result of the study could be extrapolated to the same age population. Thirdly, there are no data regarding HbA1c and oral glucose tolerance tests to determine the true prevalence of T2DM and pre-diabetes. The most important strength of the study is that it is unique with a relatively large sample size as well as precise measurement of anthropometric parameters and BP by a trained research team, which is conducted in the southern coastal region of the country. Hence, any information about the non-communicable diseases and their risk factors would be beneficial for prevention and management strategies.

 In conclusion, currently, diabetes mellitus and pre-diabetes are considered the most critical cardiovascular risk factors. Early detection of pre-diabetes can be the most effective strategy for T2DM prevention; therefore, finding the pre-diabetic individuals in an early-stage should be of high priority in prevention programs, particularly among hypertensive and dyslipidemic subjects. The significant predictors of diabetes and pre-diabetes in our study included age over 45 years, presence of obesity, HTN, hypertriglyceridemia, and living in rural areas. Accordingly, preventive strategies should be implemented in these high-risk individuals, especially in rural regions.

## References

[1] Saeedi P, Petersohn I, Salpea P, Malanda B, Karuranga S, Unwin N (2019). Global and regional diabetes prevalence estimates for 2019 and projections for 2030 and 2045: results from the International Diabetes Federation Diabetes Atlas, 9th edition. Diabetes Res Clin Pract.

[2] World Health Organization (WHO). Global Status Report on Noncommunicable Diseases 2010. WHO; 2011.

[3] Ogurtsova K, da Rocha Fernandes JD, Huang Y, Linnenkamp U, Guariguata L, Cho NH (2017). IDF Diabetes Atlas: global estimates for the prevalence of diabetes for 2015 and 2040. Diabetes Res Clin Pract.

[4] Lynch CP, Gebregziabher M, Axon RN, Hunt KE, Payne E, Egede LE (2015). Geographic and racial/ethnic variations in patterns of multimorbidity burden in patients with type 2 diabetes. J Gen Intern Med.

[5] Mirahmadizadeh A, Fathalipour M, Mokhtari AM, Zeighami S, Hassanipour S, Heiran A (2020). The prevalence of undiagnosed type 2 diabetes and prediabetes in Eastern Mediterranean region (EMRO): a systematic review and meta-analysis. Diabetes Res Clin Pract.

[6] Ebrahimi H, Emamian MH, Shariati M, Hashemi H, Fotouhi A (2016). Diabetes mellitus and its risk factors among a middle-aged population of Iran, a population-based study. Int J Diabetes Dev Ctries.

[7] Poustchi H, Eghtesad S, Kamangar F, Etemadi A, Keshtkar AA, Hekmatdoost A (2018). Prospective epidemiological research studies in Iran (the PERSIAN Cohort Study): rationale, objectives, and design. Am J Epidemiol.

[8] World Health Organization (WHO). Waist Circumference and Waist-Hip Ratio: Report of a WHO Expert Consultation, Geneva, 8-11 December 2008. WHO; 2011.

[9] James PA, Oparil S, Carter BL, Cushman WC, Dennison-Himmelfarb C, Handler J (2014). 2014 evidence-based guideline for the management of high blood pressure in adults: report from the panel members appointed to the Eighth Joint National Committee (JNC 8). JAMA.

[10] American Diabetes Association (2018). Classification and diagnosis of diabetes: standards of medical care in diabetes-2018. Diabetes Care.

[11] Jellinger PS, Smith DA, Mehta AE, Ganda O, Handelsman Y, Rodbard HW (2012). American Association of Clinical Endocrinologists’ guidelines for management of dyslipidemia and prevention of atherosclerosis. Endocr Pract.

[12] Azizi F, Khalili D, Aghajani H, Esteghamati A, Hosseinpanah F, Delavari A (2010). Appropriate waist circumference cut-off points among Iranian adults: the first report of the Iranian National Committee of Obesity. Arch Iran Med.

[13] Dare S, Mackay DF, Pell JP (2015). Relationship between smoking and obesity: a cross-sectional study of 499,504 middle-aged adults in the UK general population. PLoS One.

[14] Aadahl M, Jørgensen T (2003). Validation of a new self-report instrument for measuring physical activity. Med Sci Sports Exerc.

[15] Esteghamati A, Etemad K, Koohpayehzadeh J, Abbasi M, Meysamie A, Noshad S (2014). Trends in the prevalence of diabetes and impaired fasting glucose in association with obesity in Iran: 2005-2011. Diabetes Res Clin Pract.

[16] Akbarzadeh A, Salehi A, Molavi Vardanjani H, Poustchi H, Gandomkar A, Fattahi MR (2019). Epidemiology of adult diabetes mellitus and its correlates in pars cohort study in Southern Iran. Arch Iran Med.

[17] Esteghamati A, Gouya MM, Abbasi M, Delavari A, Alikhani S, Alaedini F (2008). Prevalence of diabetes and impaired fasting glucose in the adult population of Iran: National Survey of Risk Factors for Non-Communicable Diseases of Iran. Diabetes Care.

[18] Hadaegh F, Bozorgmanesh MR, Ghasemi A, Harati H, Saadat N, Azizi F (2008). High prevalence of undiagnosed diabetes and abnormal glucose tolerance in the Iranian urban population: Tehran Lipid and Glucose Study. BMC Public Health.

[19] Onat A, Hergenç G, Uyarel H, Can G, Ozhan H (2006). Prevalence, incidence, predictors and outcome of type 2 diabetes in Turkey. Anadolu Kardiyol Derg.

[20] Alhazmi RS, Ahmed AAB, Alshalan MH, Alfuhigi ZD, Alhazmi SF, Aldughmi AN (2017). Prevalence of diabetes mellitus and its relation with obesity in Turaif (Saudi Arabia) in 2017. Electron Physician.

[21] Taheri Tanjani P, Moradinazar M, Esmail Mottlagh M, Najafi F (2015). The prevalence of diabetes mellitus (DM) type II among Iranian elderly population and its association with other age-related diseases, 2012. Arch Gerontol Geriatr.

[22] Mirzaei M, Rahmaninan M, Mirzaei M, Nadjarzadeh A, Dehghani Tafti AA (2020). Epidemiology of diabetes mellitus, pre-diabetes, undiagnosed and uncontrolled diabetes in Central Iran: results from Yazd health study. BMC Public Health.

[23] Menke A, Casagrande S, Geiss L, Cowie CC (2015). Prevalence of and trends in diabetes among adults in the United States, 1988-2012. JAMA.

[24] Mauvais-Jarvis F (2018). Gender differences in glucose homeostasis and diabetes. Physiol Behav.

[25] Katibeh M, Hosseini S, Soleimanizad R, Manaviat MR, Kheiri B, Khabazkhoob M (2015). Prevalence and risk factors of diabetes mellitus in a central district in Islamic Republic of Iran: a population-based study on adults aged 40-80 years. East Mediterr Health J.

[26] Chiwanga FS, Njelekela MA, Diamond MB, Bajunirwe F, Guwatudde D, Nankya-Mutyoba J (2016). Urban and rural prevalence of diabetes and pre-diabetes and risk factors associated with diabetes in Tanzania and Uganda. Glob Health Action.

[27] Wang Q, Zhang X, Fang L, Guan Q, Guan L, Li Q (2018). Prevalence, awareness, treatment and control of diabetes mellitus among middle-aged and elderly people in a rural Chinese population: a cross-sectional study. PLoS One.

[28] Safari-Faramani R, Rajati F, Tavakol K, Hamzeh B, Pasdar Y, Moradinazar M (2019). Prevalence, awareness, treatment, control, and the associated factors of diabetes in an Iranian Kurdish population. J Diabetes Res.

[29] Soriguer F, Goday A, Bosch-Comas A, Bordiú E, Calle-Pascual A, Carmena R (2012). Prevalence of diabetes mellitus and impaired glucose regulation in Spain: the Di@betes Study. Diabetologia.

[30] Asadi-Lari M, Khosravi A, Nedjat S, Mansournia MA, Majdzadeh R, Mohammad K (2016). Socioeconomic status and prevalence of self-reported diabetes among adults in Tehran: results from a large population-based cross-sectional study (Urban HEART-2). J Endocrinol Invest.

[31] Pendharkar SA, Mathew J, Petrov MS (2017). Age- and sex-specific prevalence of diabetes associated with diseases of the exocrine pancreas: a population-based study. Dig Liver Dis.

[32] Saely CH, Aczel S, Koch L, Schmid F, Marte T, Huber K (2010). Diabetes as a coronary artery disease risk equivalent: before a change of paradigm?. Eur J Cardiovasc Prev Rehabil.

